
JOURNAL INFORMATION
==============================
NLM Title Abbreviation: South Med Rec
NLM Journal ID: 0027317

The Southern Medical Record

Publisher: Biomedical Journal Digitization Project

ARTICLE INFORMATION
==============================
PMCID: PMC9095004
PMID: 36024286
Article version: 1
Subjects: Editorial

So. Med. Col.

Print publication date: 1889 Dec
Volume: 19
Issue: 12
First page: 478
Last page: 478
License: This work is free of known copyright restrictions. For more information, please see PMC Back Issue Digitization.
License URL: https://creativecommons.org/publicdomain/mark/1.0/

==============================

THE MEDICAL NEWS & VISITING LIST, 1890. Thirty patients per week. Lea Brothers & Co.

This is an admirable visiting list for the physician. Revised and improved from former editions. It contains much useful memoranda and information, with table of doses. Therapeutic table calendar; ligation of arteries, incompatibles; obstetric table; weights and measures; catheter scale, etc., etc.

THE BUFFALO LITHIA SPRINGS.—We again invite attention to these excellent springs, as referred to in an advertisement in this Journal, headed *Buffalo Lithia Water*.

The profession everywhere recognize the great value of these waters, especially in kidney and bladder troubles. Dyspepsia, rheumatism, gout, etc. See the advertisement.

We had the pleasure of a call from the accommodating agent, Thomas W. Hood, in the interest of the "Maltine" Co. The samples handed us were beautiful, and of superior quality. This house and their goods stand deservedly high with the profession everywhere. Among them Maltine with Pepsin and Pancreatin; Maltine with Cascara; Maltine with Iron, Quinine and Strychnia, etc.

HENRY R. WAMPOLE & CO., 418 Arch st., Philadelphia, Pa., have been kindly handed the following beautiful samples by the gentlemanly agent of the above excellent House, to-wit: Wampole's Compound Hypophosphites; Wampole's tasteless preparation Cod Liver Oil; Wampole's concentrated extract Malt; Wampole's Syrup Hydriodic Acid; Wampole's Bromo Pyrine; Glycerine Suppositories.

THE SOUTHERN MEDICAL COLLEGE is in prosperous operation in all of its departments, both Dental and Medical. The Hospital is also in good running order, and treating all classes of patients, Surgical, Medical and gynecological.

The annual opening of the College occurs the first week in October of each year. Parties desiring information can find it by addressing a note to Dr. W. P. Nicolson, the dean of the medical department, or Dr. Wm Crenshaw, the dean of the dental department.

### SPECIAL NOTES.

**Dallas, Tex.,** June 5, 1888. Messrs. Reed & Carnrick, Gentlemen: It gives me pleasure to say that I regard your Food Preparations far superior to all others. I can point to many little ones whose lives, I feel confident, were saved by them. I have been practicing medicine in Texas for twenty-two years, have tried many other preparations, but after all I hold to yours as the old reliable; they have never disappointed me. My motive in making this statement is that others may be induced to give them a fair trial.

Yours truly, J. L. CUNNINGHAM, M. D.
